# Probiotics in Celiac Disease: Current Evidence and Future Directions

**DOI:** 10.3390/nu18193108

**Published:** 2026-09-22

**Authors:** Christopher McLaughlin, Suneeta Krishnareddy

**Affiliations:** 1 Department of Medicine, Columbia University Irving Medical Center, New York, NY 10032, USA; 2 Department of Digestive and Liver Diseases, Columbia University Irving Medical Center, New York, NY 10032, USA

**Keywords:** Celiac disease, gluten-free diet, gut microbiome, probiotics

## Abstract

Celiac disease is often associated with gut microbiome alterations, which may play a role in autoimmunity to gluten peptides and upregulation of other autoinflammatory pathways. Probiotics of various formulations have been evaluated in attempts to normalize the gut microbiome and/or improve symptoms in Celiac disease, and even to potentially prevent Celiac disease in at-risk individuals. These studies have shown mixed results at best, and conclusions about the effectiveness of probiotics to these ends are limited by the variations across studies’ probiotic formulation, dietary regimen, and clinical outcome markers. This leaves many encouraging future directions for further investigation into the potential beneficial role of probiotics on a pathophysiological and clinical level in Celiac disease.

## 1. Introduction

Celiac disease (CD) is a chronic autoimmune condition in which genetically susceptible children or adults develop an immune reaction to gluten, a dietary protein mixture including gliadin and glutenin found largely in wheat, barley, and rye [1]. Autoimmunity to gluten appears to cause disease through several mechanisms, including an adaptive immune response to the tissue transglutaminase (TTG) enzyme; in CD, TTG increases the immunogenicity of gluten peptides, activating gluten-specific T cells that damage intestinal villi, largely in the small bowel [1]. This often leads to clinical manifestations such as abdominal pain, bloating, chronic diarrhea, and malabsorption of essential vitamins and minerals, as well as chronic inflammation contributing to iron deficiency, low bone mineral density, infertility, weight loss, neuropathy, and lymphoma [2,3,4]. Although CD has various known genetic risk factors—many related to immune function [5,6,7]—many genetically at-risk individuals never develop the disease [2,3,4,5,6,7,8], suggesting that environmental factors also play a significant role in CD pathophysiology. Proposed triggers include viral and other intestinal infections, alterations in intestinal permeability, infant feeding practices, and antibiotic use [8,9,10,11]. Notably, many of these may influence, or be mediated by, the gut microbiome, which has generated growing interest in intestinal dysbiosis as a contributor to CD onset and clinical manifestations.

Previous studies have shown that colonized bacteria can eliminate immunogenic gluten peptides via their own peptidases, modulate intrinsic digestion of potentially immunogenic peptides, and even express epitopes similar to gluten peptides that may trigger host immune responses [8]. The microbiome can also influence intestinal permeability, thus affecting exposure to intestinal antigens, and release cytokines or inflammation-modulating peptides that may be either pro- or anti-inflammatory [8]. Several studies have found that CD is indeed associated with pro-inflammatory intestinal dysbiosis and suggest that gut microbiota composition may meaningfully contribute to CD development [10,12,13,14]. The balance of intestinal microbiota, therefore, may plausibly influence the development and propagation of autoinflammation in CD. This has spurred growing interest in probiotics as potential modulators of intestinal flora toward a more anti-inflammatory profile [9].

If probiotics can favorably alter the microbiome, they could play a significant therapeutic role in the management of CD. Because exposure to gluten is essential to CD pathogenesis, the only approved treatment remains strict adherence to a GFD. However, this approach has limitations: complete avoidance of gluten is financially burdensome, restrictive, psychologically challenging [12], and difficult to achieve, as many individuals unknowingly consume trace amounts of gluten [15]. Additionally, despite GFD adherence, many patients do not achieve full symptomatic or histologic recovery, and micronutrient malabsorption with secondary health consequences remains prevalent [13,14]. In a recent survey of adults with CD, approximately one-third reported using probiotics in addition to a gluten-free diet (GFD), and those who did so were more likely to report persistent symptoms despite dietary adherence [16]—indicating substantial patient interest in probiotics for additional symptom control. If probiotics can modify gut microbiota in ways that prevent CD in at-risk individuals or ameliorate symptoms and clinical consequences in those with established disease, incorporating them into treatment could help address several challenges that those with CD face today. Our aim was to summarize what the scientific literature has shown to date regarding the role of probiotics in addressing the pathophysiology and clinical manifestations of Celiac Disease.

### Search Strategy

In this narrative review, a literature search was conducted in PubMed using the terms “celiac disease AND gut microbiome,” and “celiac disease AND probiotics”, restricted to articles published in English through April 2026. These searches yielded 505 and 282 results, respectively. Additional references were identified through manual review of reference lists of included articles.

## 2. Celiac Disease Pathophysiology

In brief, CD pathophysiology is understood to be rooted in an interplay of genetic susceptibility, environmental exposure, and subsequent autoimmune response. Genetic susceptibility is largely attributable to HLA-DQ2 and HLA-DQ8, though a growing number of non-HLA loci have also been implicated—including 15 novel risk loci identified in a recent genome-wide association study of over 52,000 screened adults [17]. However, many genetically at-risk individuals never develop the disease [2,3,4,5,6,7,8], suggesting that these genetic risk factors may be necessary but not sufficient for CD to develop. An additional environmental exposure is thought to be the trigger for at-risk individuals to develop disease pathophysiology.

Among proposed environmental triggers, infant feeding practices have received particular attention. A prospective cohort study of genetically at-risk children found that specific dietary sources of gluten in early life (such as high intake of bread at 12 months and milk–cereal in the second year of life) associated with CD development—as opposed to total gluten intake alone [18]. Other potential exposures include early-life gastrointestinal and viral infections; a recent cohort analysis of 6132 genetically at-risk children followed from 3 to 48 months of age for infectious episodes, with CD autoimmunity assessed before age 4 and confirmed CD by age 14, linked infectious episodes and enterovirus exposure to subsequent disease risk [19]. Antibiotic exposure has also been proposed as a potential trigger; individuals with CD have been found to have higher odds of prior antibiotic use. However, a recent nationwide sibling analysis found this association with antibiotic use weakened substantially when comparing patients to their siblings, suggesting the relationship may at least partly reflect a higher likelihood for screening for CD in the population that presents for antibiotic prescription [20].

Mechanistically, when tissue transglutaminases deamidate gluten peptides, this increases their affinity for HLA-DQ2/DQ8 on antigen-presenting cells, thereby driving a CD4+ T cell-mediated Th1 response marked by IFN-γ and IL-21. These cytokines, together with IL-15 released by epithelial and dendritic cells, then activate cytotoxic intraepithelial lymphocytes that damage enterocytes. Additionally, innate immune pathways, including those triggered by gliadin-derived peptides and type I interferons, cause additional gut epithelial stress [21]. This cascade of autoinflammation, brought on by environmental triggers in genetically at-risk individuals, is the setting in which gut microbiota influence their inflammatory milieu.

## 3. The Role of Gut Microbiota in Celiac Disease Pathophysiology

Individuals with CD exhibit less diverse intestinal flora [9,12,22] and altered relative proportions of specific bacterial taxa compared with those without CD. Several of these changes plausibly relate to inflammatory pathways relevant to CD. *Enterobacteriaceae*, for example, are the predominantly represented bacterial family in the duodenal microbiota of CD patients [23] and contain immunostimulatory motifs that enhance IL-8, TNF-α, and IL-1β responses, thereby upregulating inflammation [24]. *Neisseria*—particularly *N. flavescens*—is also more abundant in children with active CD [10,25]; this species directly induces duodenal dendritic cells to release inflammatory cytokines, perpetuating inflammatory enteropathy [26]. Conversely, *Firmicutes* (including *Lactobacillus*) are less abundant in children with active CD [10,25,27], and their relative prevalence negatively correlates with TNF-α [28], suggesting that reductions may associate with heightened inflammatory activity. *Shigella* and *E. coli* are more prevalent in both treated and untreated CD and produce more pro-inflammatory cytokines when exposed to gliadin [29,30]. *Blautia wexlerae* appears to be less prevalent in CD [14]; this bacterium was recently shown to downregulate inflammatory cytokines in adipose tissue—a finding with plausible relevance to the autoinflammation in CD—and produce metabolites including S-adenosylmethionine, acetylcholine, and l-ornithine previously shown to have anti-inflammatory properties [31]. *Faecalibacterium prausnitzii*, an organism with known anti-inflammatory properties [32], is reduced before CD onset in at-risk children, suggesting that the relative reduction in its anti-inflammatory effects may relate to increased risk for developing CD. *Staphylococcus* (particularly *S. saprophyticus*) and *E. coli* are also more prevalent in untreated CD, and increased virulence factors have been observed in these taxa in CD [33,34,35]. Collectively, these alterations support a role for gut microbiota in CD autoinflammation.

Beyond this role in autoinflammation, other alterations in the intestinal flora of CD relate more directly to gluten metabolism. Children with CD have lower intestinal *Lactobacillus* and *Bifidobacteria* regardless of disease activity [14,34,36,37], with specific predominance of *Lactobacillus curvatus* and decreased *B. longum* [8,38]. These genera play a role in gluten metabolism and are among the bacteria with enzymes active against one of the main immunogenic gluten peptides in those with CD. *Lactobacillus* specifically appears to break down gluten into metabolites with relatively low immunogenic potential [8,9,39]. *Bifidobacteria*—particularly *B. longum*—may also stimulate the anti-inflammatory cytokine IL-10, suggesting loss of both metabolic and immunologic protection [40,41]. Their relative deficiency may, therefore, impair gluten breakdown or shift metabolism toward more immunogenic peptides.

Additional differences include increased *Bacteroides* (especially *B. fragilis*) and elevated Proteobacteria in untreated CD [10,27,34,36]. *Bacteroides* have been shown to have gliadin-specific protease activity; gliadin appears to activate innate immune responses in the intestinal epithelium in CD, and it is associated with increased intestinal permeability [8]. Altered *Bacteroides* composition may, therefore, modulate gliadin-induced inflammation. Proteobacteria—including *Enterobacteriaceae*—not only increase intestinal permeability [8,42] but also metabolize gluten into highly pro-inflammatory products [8,12]. Together, these findings suggest that dysbiosis may influence gluten metabolism, which may further relate to CD pathophysiology.

Still, other differences in the intestinal flora in CD have been observed without a clear suggested relation to CD pathogenesis. *Haemophilus*, *Clostridium*, *Methylobacterium*, and *Mycobacterium* are also more prevalent in CD [10,22], some of which have increased prevalence in those with Crohn’s disease and ulcerative colitis [43], though their precise roles remain unclear.

Despite treatment with a GFD, the gut microbiota often remains abnormal in CD. Several studies found that despite maintaining a GFD, patients with CD continued to exhibit reduced flora diversity and low *Bifidobacteria* and *Lactobacilli* compared with controls [14,34,44,45,46]. Some evidence even suggests that a GFD may further reduce these genera in both CD and non-CD individuals [47,48], potentially due to limited prebiotic content [49]. Thus, a GFD alone may not correct dysbiosis, with implications for a possible clinical benefit of probiotics if microbial changes influence gluten metabolism and autoinflammation.

## 4. Composition of Probiotics

Given the alterations in the intestinal microbiome of those with CD described above, several probiotic formulations have been developed—largely targeting the specific genera most consistently found to be deficient. Many utilize *Lactobacillus*, which is deficient in CD and appears to reduce intestinal permeability, downregulate inflammation, and metabolize gluten to less immunogenic products. These probiotics specifically include *L. plantarum* HEAL9 and *L. paracasei* 8700:2 in a 1:1 ratio [47,50,51,52]. Others target *Bifidobacteria*, also reduced in CD and shown to have various anti-inflammatory effects. Current formulations include *B. breve* BR03 and B632 (1:1, 2 × 10^9^ CFU), *B. longum* CECT 7347 (10^9^ CFU), and *B. infantis* (2 × 10^9^ CFU) [28,38,53,54,55,56]. Combination probiotics containing both genera have also been developed, with some evidence suggesting enhanced anti-inflammatory and gluten-metabolizing activity when used together [57]. These include mixtures of *L. casei*, *L. plantarum*, *L. rhamnosus*, *B. animalis*, *B. breve*, and *B. lactis* strains [58,59,60] and proprietary blends of *B. breve*, *B. longum*, *B. infantis*, *L. acidophilus*, *L. plantarum*, *L. paracasei*, *L. delbrueckii*, and *Streptococcus thermophilus* [61]. Some studies have used yogurt as a natural source of *L. acidophilus* and *B. lactis*, though additional probiotic strains present were not assessed [62].

Few studies have explored probiotics containing bacterial genera beyond *Lactobacillus* and *Bifidobacteria*. One proprietary blend included *Bifidobacteria* and *Clostridium butyricum*, previously shown to similarly reduce intestinal levels of inflammatory cytokines [63,64].

Although various bacteria have been shown to exist in relatively different proportions among the CD gut microbiome, probiotics studied to date predominantly incorporate *Lactobacillus* and/or *Bifidobacteria*. While these organisms are indeed deficient in CD and plausibly influence gluten metabolism and immune pathways, causal links are not yet established. Their associations with CD-related mechanisms do, however, support ongoing research into their therapeutic potential.

## 5. The Effect of Probiotics on Gut Microbiota in Celiac Disease

Because the GFD does not appear to fully normalize the gut microbiota, multiple studies have evaluated whether probiotics can do so (Table 1). Results have been mixed.

Oscarsson et al. randomized 78 children with CD autoimmunity to receive either *L. plantarum* HEAL9 + *L. paracasei* 8700:2 (10^10^ CFU/g) or maltodextrin placebo daily for 6 months while continuing a gluten-containing diet, with avoidance of other probiotic sources. After 6 months, the probiotic group showed increased *Lactobacillus*, compared to a decrease in the placebo group; the probiotic group also showed decreased *Lactococcus* and *Bifidobacterium* and stable *Streptococcus*, which declined in the placebo group. No differences in other taxa or in alpha/beta diversity were observed [50]. Other groups have utilized probiotics with isolated *Bifidobacteria*. Quagliariello et al. randomized 40 children ages 1–19 on a GFD to *B. breve* BR03 and B632 or placebo. Children with CD initially had lower *Actinobacteria* and a lower *Firmicutes/Bacteroides* ratio than children without CD, but both normalized only in those receiving the probiotic after 3 months. However, probiotics did not raise Euryarchaeota or Proteobacteria to control levels [54]. Olivares et al. randomized 36 children ages 2–17 with newly diagnosed CD to GFD plus *B. longum* CECT 7347 or placebo for 3 months with dietary avoidance of other probiotics. Groups showed largely comparable gut microbiota; the probiotic-receiving group had significantly reduced *B. fragilis*, but no significant difference was otherwise found regarding total bacteria, *Enterobacteriaceae*, *Lactobacillus*, *Bifidobacterium*, *C. coccoides*, or *C. leptum* [55]. Overall, probiotics containing predominantly either *Lactobacillus* or *Bifidobacterium* have shown minimal effect in normalizing gut microbiota diversity or normalizing proportions of many otherwise altered bacterial populations.

Studies of combination probiotics have also shown limited effects. Harnett et al. randomized 45 adults with CD and persistent symptoms despite at least 1 year of GFD to receive either a multi-strain probiotic of *Streptococcus thermophilus*, *Bifidobacterium breve*, *B. longum*, *B. infantis*, *L. acidophilus*, *L. plantarum*, *L. paracasei*, and *L. delbrueckii* subsp. *bulgaricus* or placebo for 12 weeks. Nutritional monitoring of dietary gluten but not probiotic-containing foods was done, though participants who had taken antibiotics in the preceding month of study initiation were excluded. No significant microbiota differences were observed [61]. Lionetti et al. similarly randomized 96 newly diagnosed children ages 2–16 to GFD plus a combination probiotic of *L. casei* LMG 101/37 P-17504, *L. plantarum* CECT 4528, *B. animalis* subsp. *lactis* Bi1 LMG P-17502, *B. breve* Bbr8 LMG P-17501, and *B. breve* Bl10 LMG P-17500 or placebo for 12 weeks. No explicit monitoring of diet or other probiotic intake was done, though patients with recent antibiotic or proton-pump inhibitor use were excluded. No significant effects on microbiota diversity or abundance were observed [59]. Martinello et al. gave yogurt containing *L. acidophilus* and *B. lactis* to 14 adults with asymptomatic CD on GFD who had not eaten any foods with probiotics for at least 3 months; reduced baseline *Bifidobacteria* levels did not normalize [62]. Francavilla et al., however, found that a similar combination probiotic containing *L. casei* LMG 101/37 P-17504 (5 × 10^9^ CFU/sachet), *L. plantarum* CECT 4528 (5 × 10^9^ CFU/sachet), *B. animalis* subsp. *lactis* Bi1 LMG P-17502 (10 × 10^9^ CFU/sachet), *B. breve* Bbr8 LMG P-17501 (10 × 10^9^ CFU/sachet), and *B. breve* Bl10 LMG P-17500 (10 × 10^9^ CFU/sachet) significantly increased *Bifidobacterium*, *Lactobacillus*, *Lactococcus*, *Streptococcus*, and *Staphylococcus* after 6 weeks of once daily use among 109 adults with CD and persistent IBS symptoms, though no changes in diversity or overall genus-level relative abundance occurred [58].

Taken together, these studies provide mixed evidence regarding the ability of probiotics to affect the gut microbiota in CD. Studies have largely utilized small samples with inconsistent control over diet and gluten intake, restrictions on other probiotic intake, and probiotic formulations. Despite this, studies to date have largely found that these probiotics may have some ability to increase the specific abundance of *Lactobacillus* or *Bifidobacterium* among some other relatively deficient genera depending on the probiotic formulation, but have minimal effect on overall gut microbiota diversity.

## 6. Evidence of Clinical Benefit from Probiotic Use in Celiac Disease

### 6.1. Benefit in Previously Diagnosed Celiac Disease

A central question for symptomatic CD is whether probiotics—regardless of their effects on the gut microbiota—can provide clinical benefit. Existing studies show mixed results (Table 1).

*Lactobacillus*-based studies include Håkansson et al., who used the Oscarsson et al. protocol described above in 78 children ages 2–12 on gluten; subtle lymphocyte differences were found without group differences in levels of TTG-IgA, the autoantibody directed against tissue transglutaminase seen in CD [47]. Jenickova et al. also utilized this protocol and identified altered fecal amino acids and higher 4-hydroxyphenylacetate potentially indicative of antioxidative function, but there was no clear association with clinical outcomes or symptom benefit [52].

Studies of *Bifidobacteria* have shown mixed results. The Olivares et al. study described above found no group differences in diarrhea, constipation, or pain between children receiving probiotics or placebo; height increased more with probiotics, but weight and most immune markers were similar [55]. Smecuol et al. randomized 22 adults with CD to take either two capsules of *B. infantis* (2 × 10^9^ CFU/capsule) or placebo before each meal while on a gluten-containing diet and while restricting other probiotic intake. In this study, both groups continued to eat a set amount of gluten per day and were restricted from taking additional probiotics or probiotic-containing foods. After 3 weeks, those receiving the probiotic reported reduced indigestion and constipation, with no TTG-IgA or intestinal permeability change [56]. A follow-up of this probiotic in 12 symptomatic adults now on GFD found improved Celiac Symptom Index (CSI) scores [38]. Pinto-Sánchez et al. showed reduced duodenal macrophages and decreased Paneth cells and α-defensin-5—markers of innate immunity—after 3 weeks of *B. infantis* compared to placebo among those not on GFD, though this benefit was less than that seen after simply 1 year of GFD alone [65]. Klemenak et al. randomized 49 children ages 1–19 on GFD to 1:1 *B. breve* BR03 and B632; participants were excluded if they took antibiotics within 1 month of the study, but no specific monitoring of diet or other probiotics was done. After 3 months, probiotics reduced serum TNF-α compared to placebo, though mainly in children on GFD for over 1 year; clinical symptoms were not assessed [53]. Ali et al. randomized 170 newly diagnosed children ages 8–10 to GFD with or without Gutcare™ (500 mg sachet of *C. butyricum* and *Bifidobacterium*) twice daily; no dietary monitoring was performed. After 28 days, both groups had decreased stool frequency, but probiotics yielded a significantly greater reduction [63].

Overall, these studies of probiotics containing *Lactobacillus* or *Bifidobacterium* alone showed mixed results regarding their benefit for clinical symptoms or inflammatory markers in CD. While some formulations may show some benefit in certain circumstances, these studies are difficult to generalize given their small samples, mixed inclusion criteria, minimal dietary standardization, and highly varied clinical outcome markers.

Studies investigating combination probiotics also vary (Table 1). The Francavilla et al. study described above observed significant improvement in IBS-like symptoms in 109 adults taking a 5-strain *Lactobacillus/Bifidobacterium* probiotic compared to placebo [58]. However, Lionetti et al. evaluated a very similar probiotic strain and found no differences in GI symptoms, TTG-IgA levels, ferritin, hemoglobin, vitamin D, or fecal calprotectin after 6 months of use in newly diagnosed children starting a GFD, though the probiotic group did show a greater BMI-Z increase [59]. Jesus et al. used a slightly different combination probiotic of *B. lactis* and *L. rhamnosus* with adults with CD who may or may not have been adherent to GFD, randomized to receive either probiotic or placebo daily for 90 days. Here, the treatment group did show a significant improvement compared to placebo in symptoms measured by the Gastrointestinal Symptom Rating Scale (GSRS), as well as in emotional well-being as measured by the CD-QOL [60]. However, Harnett et al., as described above, found no microbiota or symptom (diarrhea, abdominal pain, or emotional disturbance) effects in symptomatic adults on GFD using a similar combination probiotic for 12 weeks [61]. These studies also generally provide mixed evidence to suggest combination probiotics of *Lactobacillus* and *Bifidobacterium* provide clinical benefit regarding symptom relief, disease activity, or secondary health consequences for patients with CD.

### 6.2. Primary Prevention of Celiac Disease

A recent in vitro study by Tran et al. showed promising results utilizing a novel strain of *Bacteroides vulgatus*—previously found to be decreased in abundance in the gut microbiota of children at risk of CD who went on to develop CD compared to those at risk who did not [68]. Tran et al. found that gut epithelium from these at-risk children underwent increased epithelial cell death and permeability with an associated inflammatory response in response to gliadin exposure, but this process was mitigated upon exposure to *B. vulgatus*, suggesting that this probiotic strain may play a role in reducing the inflammatory effect of gliadin and preventing development of CD in at-risk individuals.

Only Uusitalo et al. have performed an in vivo study of the role of probiotics in prevention of CD in high-risk individuals (Table 1). Among 6520 children with at-risk HLA genotypes, exposure in the first year of life to probiotics largely consisting of *Lactobacillus* via dietary supplement or infant formula did not associate with a difference in developing CD by age 15. Interestingly, probiotic use was linked to slightly increased odds of persistently positive TTG antibodies but not confirmed CD [66]. Future studies on this topic should standardize probiotic formulations and timing of exposure and incorporate both serologic and histologic endpoints to more definitively characterize a relationship between early probiotic use and CD development.

### 6.3. Role of Probiotics in Gluten Degradation

Beyond their effects on inflammation and overall microbiome composition, an emerging line of evidence has examined probiotics’ direct capacity to metabolize gluten itself. Certain bacterial genera possess peptidases capable of degrading immunogenic gluten peptides; *Lactobacillus* has particularly been shown to break gluten down into metabolites of comparatively low immunogenicity [8,9,39]. In vitro work with a combination probiotic containing multiple *Lactobacillus* and *Bifidobacterium* strains currently marketed as “Visbiome” in North America or “CdS22” in Europe (at the time of publication referred to as “VSL#3”) can also hydrolyze gliadin polypeptides implicated in CD pathogenesis, suggesting that gluten-metabolizing activity may be enhanced when complementary strains are combined [57]. More recently, a multi-strain probiotic developed specifically for its in vitro gluten-degrading properties was shown to significantly reduce fecal gluten content in healthy volunteers following a period of gluten reintroduction [67] (Table 1). These findings highlight the ability of certain probiotics to enhance gluten degradation beyond solely altering microbial composition or inflammation. While such activity does not necessitate clinical benefit, it may represent a distinct and complementary therapeutic strategy warranting further investigation in this population.

## 7. Future Directions

Whereas earlier systematic reviews of probiotics in CD were limited to RCTs assessing GI symptoms and QOL through 2019 [9,69], here we incorporate a more recent body of evidence. Newer trials of *Lactobacillus*-based formulations extended prior microbiome-focused findings to fecal metabolome outcomes, though without clear symptom correlates [50,51,52]. A follow-up study of *B. infantis* now adds evidence of symptom improvement for adults on long-term GFD [38]. New combination probiotics have also been assessed, including a *B. lactis*/*L. rhamnosus* combination showing improvement in GI symptoms and emotional well-being [60] and a *C. butyricum*/*Bifidobacterium* formulation [58]; clinical benefit remains mixed. New studies also evaluate a role in primary prevention, including a promising in vitro protective effect against gut epithelial damage and inflammation by *B. vulgatus* after gliadin exposure in at-risk children [68], as well as a large cohort study showing that early-life probiotic exposure in genetically at-risk children did not reduce CD incidence and was instead associated with a slight increase in persistent TTG antibody positivity [66]. Recent studies also highlight a newly identified gluten-degrading multi-strain probiotic shown to significantly reduce fecal gluten content in healthy volunteers [67]—a promising approach not yet tested in CD but that represents a meaningfully different therapeutic rationale from the *Lactobacillus*/*Bifidobacterium*-centric probiotics studied to date.

Although intestinal dysbiosis in CD is well described, causal mechanisms in CD pathogenesis or its related inflammatory consequences remain unproven. As the GFD alone only partially normalizes intestinal flora, there is potential space for probiotics to further improve the balance of gut microbiota, which may, in turn, provide clinical benefit. Future work should evaluate broader formulations targeting taxa consistently altered in CD aside from *Lactobacillus* and *Bifidobacterium* and assess outcomes across not only inflammation and symptoms but also nutritional markers and their secondary clinical consequences. Alterations of the microbiome in CD by alternative means, specifically fecal microbiota transplant, are also of interest and warrant further investigation. One case report describes a patient with refractory CD who had both complete resolution of clinical symptoms and normalization of duodenal villi following a fecal microbiota transplant for recurrent *Clostridium difficile* infection [70]. Although subsequent studies have not been done to assess causality for such a therapy, this may represent a promising avenue of microbiota alteration that could provide clinical benefit. Future studies should also investigate the role of probiotics in treatment for refractory CD, as this population is in particular need of additional treatment options, but to date, the role of probiotics for this population has not been specifically investigated. Additionally, only one study has investigated the role of probiotics in CD prevention, which remains an important space for future study. Recent work by Nikoloudaki et al. tested a gluten-degrading multi-strain probiotic of *Lactiplantibacillus plantarum DSM33363*, *Lactiplantibacillus plantarum*, *Lacticaseibacillus paracasei DSM33373*, *Limosilactobacillus reuteri DSM33374*, *Bacillus megaterium*, *Bacillus pumilus DSM33297*, and *Bacillus pumilus DSM33355*, developed based on in vitro gluten degradation abilities, in 70 healthy volunteers on a controlled Mediterranean diet with gluten withdrawal and reintroduction; despite unchanged diversity, fecal gluten was significantly reduced after 32 days [66]. This formulation has not been tested in CD but suggests potential for gluten metabolism-targeted strategies that could benefit at-risk individuals.

## 8. Limitations

It remains difficult to draw meaningful conclusions regarding the role of probiotics in treatment and prevention of CD for several reasons. The studies outlined above are widely heterogeneous regarding the probiotic strains utilized, dietary control implemented, duration of probiotic utilization, restriction of other probiotics, age ranges even among pediatric samples, and time on GFD. Outcome measures also vary widely, and there is inconsistency in whether outcomes relating to pathophysiologic markers correspond to clinical benefits in the patient population. Many of these studies utilize small samples, and some utilize proprietary blends of probiotics that may not be broadly available. Studies to date also vary widely in follow-up time course; it remains unclear whether many of the physiologic and/or clinical benefits persist over time. These studies also do not investigate long-term safety data.

Our study itself additionally has limitations. Our search strategy was not exhaustive, and the nature of our narrative review may leave additional studies with relevance to this topic unexplored.

## 9. Conclusions

Probiotic use remains common among symptomatic individuals on GFD [15]. Clearly, there is an unmet need among many in this population to provide greater symptomatic benefit than the only approved treatment allows. While the supporting data behind probiotics’ ability to modulate the microbiome, reduce inflammation, or improve clinical symptoms is mixed, it will be important to continue to develop and evaluate these already popular supplements to further investigate the unexplored gaps in our current understanding of them and to help improve the health and well-being of this population.

## Figures and Tables

**Table 1:** Summary of in vivo studies of probiotics in celiac disease.

Author (et al.)	Year	Design	Population	Intervention	Key Results
Section 5. The Effect of Probiotics on Gut Microbiota in Celiac Disease					
Oscarsson [50]	2021	RCT, placebo-controlled	78 children ages 3–7 with CD autoimmunity, continuing gluten-containing diet	*L. plantarum* HEAL9 + *L. paracasei* 8700:2 (10^10^ CFU/g) vs. maltodextrin placebo, 6 months	Increased *Lactobacillus* vs. placebo; decreased *Lactococcus* and *Bifidobacterium*; no diversity differences
Quagliariello [54]	2016	RCT, placebo-controlled	40 children ages 1–19 with CD on GFD	*B. breve* BR03 + B632 vs. placebo, 3 months	Normalized Actinobacteria and *Firmicutes*/*Bacteroides* ratio with probiotic; Euryarchaeota/Proteobacteria unchanged
Olivares [55]	2014	RCT, placebo-controlled	36 children ages 2–17 with newly diagnosed CD	*B. longum* CECT 7347 + GFD vs. placebo, 3 months	Reduced *B. fragilis* with probiotic; no other microbiome differences; no symptom differences; greater height gain with probiotic
Harnett [61]	2016	RCT, placebo-controlled	45 adults with CD and persistent symptoms despite ≥ 1 year GFD	Multi-strain probiotic (*S. thermophilus*, *B. breve*, *B. longum*, *B. infantis*, *L. acidophilus*, *L. plantarum*, *L. paracasei*, *L. delbrueckii* subsp. *bulgaricus*) vs. placebo, 12 weeks	No microbiome or symptom differences vs. placebo
Lionetti [59]	2023	RCT, placebo-controlled	96 newly diagnosed children ages 2–16	Combination probiotic (*L. casei*, *L. plantarum*, *B. animalis* subsp. *lactis*, *B. breve* [2 strains]) vs. placebo, 12 weeks	No differences in microbiome, GI symptoms, or labs vs. placebo; greater BMI-Z increase with probiotic
Martinello [62]	2017	Open label	14 adults with asymptomatic CD on GFD, no probiotic foods for ≥3 months prior	Yogurt containing *L. acidophilus* and *B. lactis*	*Bifidobacteria* levels remained low
Francavilla [58]	2019	RCT, placebo-controlled	109 adults with CD and persistent IBS-type symptoms	Combination probiotic (*L. casei*, *L. plantarum*, *B. animalis* subsp. *lactis*, *B. breve* [2 strains]) vs. placebo, 6 weeks	Increased *Bifidobacterium*, *Lactobacillus*, *Lactococcus*, *Streptococcus*, *Staphylococcus*; improved IBS-like symptoms vs. placebo
Section 6.1. Benefit in Previously Diagnosed Celiac Disease					
Håkansson [47]	2019	RCT, placebo-controlled	78 children ages 2–12 with CD autoimmunity, on gluten	*L. plantarum* HEAL9 + *L. paracasei* 8700:2 vs. placebo, 6 months	Subtle lymphocyte differences found; no group differences in TTG-IgA
Jenickova [52]	2023	RCT, placebo-controlled	78 children ages 2–11 with CD autoimmunity, on gluten	*L. plantarum* HEAL9 + *L. paracasei* 8700:2 vs. placebo, 6 months	Altered fecal amino acids; no clinical or symptom association
Smecuol [56]	2013	RCT, placebo-controlled	22 adults with CD, gluten-containing diet, other probiotics restricted	*B. infantis* (2 × 10^9^ CFU/capsule, 2 capsules before each meal) vs. placebo, 3 weeks	Reduced indigestion and constipation; no change in TTG-IgA or intestinal permeability
Smecuol [38]	2020	RCT, placebo-controlled	12 symptomatic adults, on GFD	*B. infantis* (2 × 10^9^ CFU/capsule, 2 capsules before each meal) vs. placebo, 3 weeks	Improved Celiac Symptom Index (CSI) scores
Pinto-Sánchez [65]	2017	Open label	41 adults with CD; 38 on gluten, 5 on GFD	*B. infantis* (2 × 10^9^ CFU/capsule) vs. GFD	Reduced duodenal macrophages and Paneth cells/α-defensin-5; less benefit than 1-year GFD alone
Klemenak [53]	2015	RCT, placebo-controlled	49 children ages 1–19 with CD on GFD	*B. breve* BR03 + B632 (1:1) vs. placebo, 3 months	Reduced serum TNF-α vs. placebo, mainly in children on GFD > 1 year
Ali [63]	2022	RCT	170 newly diagnosed children ages 8–10	GFD + Gutcare™ (*C. butyricum* + *Bifidobacterium*, 500 mg sachet, twice daily) vs. GFD alone, 28 days	Greater reduction in stool frequency vs. GFD alone
Jesus [60]	2025	RCT, placebo-controlled	Adults with CD, variable GFD adherence	*B. lactis* + *L. rhamnosus* vs. placebo, 90 days	Significant improvement in GSRS symptom scores and CD-QOL emotional well-being vs. placebo
Section 6.2. Primary Prevention of Celiac Disease					
Uusitalo [66]	2019	Longitudinal cohort	6520 children ages 0–1 with at-risk HLA genotypes; CD development assessed by age 15	Probiotic exposure (largely *Lactobacillus*) via dietary supplement or infant formula in first year of life	No association with CD by age 15; slightly increased odds of persistent TTG positivity
Section 6.3. Role of Probiotics in Gluten Degradation					
Nikoloudaki [67]	2024	RCT, placebo-controlled	70 adults without CD on controlled Mediterranean diet with gluten withdrawal/reintroduction	Multi-strain gluten-degrading probiotic (7 named strains) vs. placebo, 32 days	Fecal gluten significantly reduced; diversity unchanged

## Data Availability

No new data were created or analyzed in this study. Data sharing is not applicable to this article.
